# Endogenous Bornavirus‐Like Nucleoprotein 1 Regulates Cellular Processes and DNA Double‐Strand Breaks in U‐87 MG Glioblastoma Cells

**DOI:** 10.1002/cnr2.70680

**Published:** 2026-09-04

**Authors:** Jing Liang, Dongdong Zeng, Yujie Guo, Cuiyuan Jing, Lin Tang, Zhanpeng Luo, Peng He

**Affiliations:** ^1^ Department of Operating Room The Affiliated Hospital of Southwest Medical University Luzhou China; ^2^ Department of Biobank The Affiliated Hospital of Southwest Medical University Luzhou China; ^3^ NHC Key Laboratory of Diagnosis and Treatment on Brain Functional Diseases The First Affiliated Hospital of Chongqing Medical University Chongqing China; ^4^ Chongqing Blood Center Chongqing China

**Keywords:** ATM, BRCA1, DNA double‐strand break, DSB damage repair, endogenous bornavirus‐like nucleoprotein 1, glioblastoma

## Abstract

**Background:**

Glioblastoma (GBM) is a common brain cancer with a poor prognosis and a high recurrence rate. DNA damage repair plays a crucial role in GBM carcinogenesis and treatment resistance. Human endogenous bornavirus‐like nucleoprotein 1 (EBLN1) regulates cellular processes and genetic stability. However, the biological role of EBLN1 in GBM remains unclear.

**Aims:**

This study aims to determine the role of EBLN1 in the regulation of cellular processes and DNA double‐strand breaks (DSBs) in GBM and to investigate the underlying molecular mechanisms.

**Methods and Results:**

U‐87 MG GBM cells were infected with an shRNA‐expressing lentivirus to knock down EBLN1. Then, cell proliferation and apoptosis were evaluated. The levels of DSBs, as well as the activities of two key DSB repair regulators—ataxia telangiectasia mutated (ATM) kinase and breast cancer Type 1 susceptibility protein (BRCA1)—were detected. Furthermore, after reactivating ATM, cellular processes and the levels of DSBs were re‐detected to verify the mechanisms underlying EBLN1‐mediated DNA repair regulation in GBM. EBLN1‐silenced U‐87 MG cells displayed a significant reduction in cell growth as early as the second day postinfection (*p* < 0.05) and a higher apoptosis ratio (*p* < 0.001). After EBLN1‐silencing, U‐87 MG cells exhibited a remarkable increase in the expression of γH2AX (*p* < 0.001) and significantly lower expression levels of ATM and BRCA1 (*p* < 0.001). After reactivation of ATM in EBLN1‐silenced U‐87 MG cells, DSB levels in the treated cells were significantly decreased (*p* < 0.001). Meanwhile, cell proliferation was significantly increased (*p* < 0.001), while apoptosis was markedly suppressed (*p* < 0.001).

**Conclusion:**

This study uncovers a novel molecular mechanism that EBLN1 regulates cellular processes via modulating DNA double‐strand break repair in glioblastoma cells, providing a new perspective for understanding the pathogenesis of GBM.

## 1 Introduction

Glioblastoma (GBM) is the most aggressive and invasive primary brain cancer in adults, accounting for nearly half (49%) of all malignant brain and other CNS tumors [1, 2]. Despite the combination of surgical resection, chemotherapy, and radiotherapy, the prognosis of GBM is still poor, with the 5‐year survival rate less than 10% [3, 4]. Emerging evidence indicates that DNA damage response (DDR) plays a complex and critical role in GBM progression and treatment resistance [5, 6].

DDR is a sophisticated network that detects and repairs DNA damage and influences various cellular processes, including cell proliferation and apoptosis. In the occurrence and development of GBM, DDR exhibits double‐edged sword functions. On the one hand, an appropriate DDR contributes to genetic stability against GBM occurrence. However, in developed GBM, DDR can repair the DNA damage caused by chemotherapy and radiotherapy, leading to tumor survival [7, 8]. However, the underlying mechanisms are not fully illuminated. Therefore, further exploration into the regulatory mechanism of DDR will be helpful in understanding and treating GBM.

Endogenous bornavirus‐like nucleoprotein elements (EBLNs) are formed by the integration of the virus genes into the host genome during ancient bornavirus infection [9, 10]. EBLNs show homology to the nucleoprotein gene of Borna disease virus 1 (BoDV‐1), a zoonotic virus that infects humans and causes fatal encephalitis. The functions of EBLNs are yet unclear. Only a few studies indicate that EBLNs may function as protein‐coding genes, noncoding RNAs (ncRNAs), or pseudogenes to regulate cellular behaviors [11, 12, 13, 14]. Previous work has reported that human EBLN1, one type of EBLNs, plays an important role in regulating cell proliferation, cell cycle progression, and apoptosis in oligodendroglia (OL) cells [11]. Another study demonstrates that depletion of EBLN1 leads to increased DNA double‐strand breaks (DSBs)—the most severe type of DNA damage—leading to cell cycle arrest in human cancer cells [15]. Furthermore, recent studies have shown that BoDV‐1 infection or expression of its N or P proteins could increase primary neuronal DSBs [16, 17]. These results confirm the crucial role of EBLN1 in regulating cellular processes and DSB repair. However, it remains uncertain whether EBLN1 has a similar function in GBM cells and whether there is a relationship between EBLN1‐regulated DSB repair and alterations of cellular processes.

In this study, we show that EBLN1 silencing inhibits cell proliferation and induces cell apoptosis in U‐87 MG GBM cells. Furthermore, silencing of EBLN1 increases DSBs and decreases the activities of ataxia telangiectasia mutated (ATM) kinase and breast cancer Type 1 susceptibility protein (BRCA1), two crucial protein kinases involved in DSB repair pathways. Our results demonstrate that EBLN1 regulates cellular biological processes by influencing DSB repair in GBM cells. This work not only enriches the biological functions of EBLN1 but also provides a new molecular mechanism for GBM.

## 2 Materials and Methods

### 2.1 Cell Culture and Reagents

The U‐87 MG cells were purchased from the Chinese Type Culture Collection (CTCC, China) and cultured in DMEM (BasalMedia, Shanghai, China), containing 5% FBS (Solarbio, China), 1% penicillin, and 1% streptomycin. Cells were incubated at 37°C in a 5% CO_2_ incubator. The U‐87 MG cells were free of mycoplasma contamination, as determined by the Mycoplasma Colorimetric LAMP Assay Kit (Beyotime Biotechnology, China). The ATM activator chloroquine (Chq) was purchased from Selleck Chemicals (Shanghai, China).

### 2.2 EBLN1 shRNA‐Expressing Lentiviral Vector and Infection

The lentiviral vectors were constructed by OBiO Technology (Shanghai)Corp. Ltd. The EBLN1 shRNA targeted the sequence of EBLN1 mRNA (NM_001199938): 5′‐GCCTCCAAGTCAGA GACA‐3′ [11]. For lentiviral packaging, the shRNA was cloned into the pSLenti‐U6‐shRNA plasmid and then co‐transfected into 293T cells with Gag/pol expression plasmid, VSV‐G expression plasmid, and Rev expression plasmid. The EBLN1 shRNA‐expressing lentiviral vector was termed pSLenti‐EBLN1‐shRNA; the negative control lentiviral vector was termed pSLenti‐EBLN1‐Neg. The U‐87 MG cells were divided into three groups: uninfected group (CON); negative control group (NC), infected with pSLenti‐EBLN1‐Neg; EBLN1‐silenced group (KD), infected with pSLenti‐EBLN1‐shRNA. After 72 h postinfection, infection efficiency was measured.

### 2.3 Quantitative Reverse Transcription‐Polymerase Chain Reaction (qRT‐PCR)

The relative expression of EBLN1 was tested using qRT‐PCR according to previous work [11]. Total RNA was extracted using the RNAeasy RNA extraction kit (Beyotime Biotechnology, China) according to the manual. The quality and quantity of RNA were assessed by a NanoDrop One spectrophotometer (Thermo Fisher Scientific, USA). QRT‐PCR was performed using SYBR Green Supermix (TaKaRa, Japan) following the manufacturer's instructions on an SLAN‐96S real‐time PCR instrument (Hongshi Medical Technology, China). The PCR primers used for detecting the EBLN1 gene were: 5′‐ACCTAGCAACAGCAGCAAACTA‐3′ (forward); 5′‐CAAATCCCGA AATCCCATAAC‐3′ (reverse). For the reference gene GAPDH, the primers were: 5′‐GGTCTCCTCTGACTTCAACA‐3′ (forward); 5′‐AGCCAAATTCGTTGTCATAC‐3′ (reverse). PCR cycling conditions were 95°C for 2 min, followed by 40 cycles of 95°C for 15 s and 60°C for 30 s. The experiments were repeated nine times. The relative expression of EBLN1 was calculated using the 2^−∆∆Ct^ method.

### 2.4 Cell Proliferation

Cell proliferation was analyzed as described previously [11]. The Cell Counting Kit‐8 was purchased from Beyotime Biotechnology (Shanghai, China). Briefly, U‐87 MG cells were plated in 96‐well plates at a density of 2000 cells per well in 100 μL of culture medium. From 1 to 5 days after plating, 10 μL of CCK‐8 solution was added to each well and incubated for 2 h at 37°C. Then, the absorbance was measured at 450 nm using a Multiskan FC spectrophotometer (Thermo Fisher Scientific, USA). The OD450 ratio (OD450/fold) was calculated using the OD450 value of Day 1 as a reference.

### 2.5 Apoptosis

Cell apoptosis was measured by annexin V‐APC staining (Beyotime Biotechnology, China). After 96 h of incubation, U‐87 MG cells were washed twice with cold PBS, then digested with 0.25% trypsin and collected by centrifugation at 3000 rpm for 5 min. The collected cells were washed three times with prechilled PBS and resuspended in 1 × binding buffer at a concentration of 5 × 10^5^ cells/mL. Add 5 μL of Annexin V‐APC to 100 μL of the cell suspension. After gentle mixing, the cells were incubated for 15 min at room temperature in the dark. Then, 100 μL of 1 × binding buffer was added to each assay. Finally, the samples were analyzed by flow cytometry (BD Biosciences, San Jose, California, USA) within 1 h.

### 2.6 Enzyme‐Linked Immunosorbent Assay (ELISA)

ELISA was employed to measure the concentrations of phosphorylated histone H2AX (γH2AX), ATM, and BRCA1. The human γH2AX ELISA kit and BRCA1 ELISA kit were purchased from Fine Biotech Co. Ltd. (Wuhan, China). The human phospho‐ATM (S1981) ELISA kit was purchased from Enzyme‐linked Biotechnology Co. Ltd. (Shanghai, China). After 96 h of incubation, the U‐87 MG cells in each group were gently washed with prechilled PBS, then digested with 0.25% trypsin and collected by centrifugation at 3000 rpm for 5 min. The collected cells were washed three times with prechilled PBS and resuspended in 200 μL of PBS per 1 × 10^6^ cells. Cells were lysed by repeated freezing and thawing. The lysate was centrifuged at 3000 rpm for 20 min at 4°C. The supernatant was used for ELISA assays following the manufacturer's instructions.

### 2.7 Statistical Analysis

All experiments were repeated three times, except for the cell Proliferation assay, which was repeated five times. Statistical analysis was performed using SPSS software version 26.0 (IBM Corporation, New York City, New York, USA). All quantitative data were expressed as the mean ± standard deviation (SD). Student's *t*‐test and ANOVA were used to analyze the statistical differences between the groups. A value of *p* < 0.05 was considered statistically significant.

## 3 Results

### 3.1 Expression of EBLN1 mRNA Was Effectively Depressed in U‐87 MG Cells

**FIGURE 1:**
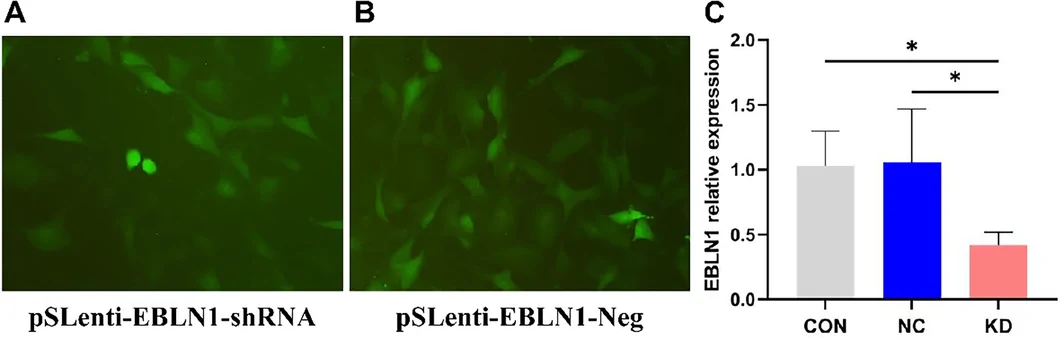
EBLN1 mRNA was effectively knocked down in U‐87 MG cells infected with the EBLN1 shRNA‐expressing lentivirus. (A) Infection efficiencies of pSLenti‐EBLN1 shRNA‐expressing lentivirus (200×); (B) infection efficiencies of pSLenti‐EBLN1‐Neg‐expressing lentivirus (200×); (C) relative expression of EBLN1 mRNA by qRT‐PCR. CON, uninfected group; KD, EBLN1‐silenced group (infected with pSLenti‐EBLN1‐shRNA); NC, negative control group (infected with pSLenti‐EBLN1‐Neg). *p < 0.001.

The U‐87 MG cells were infected with EBLN1 shRNA‐expressing lentivirus (pSLenti‐EBLN1‐shRNA) and negative‐expressing lentivirus (pSLenti‐EBLN1‐Neg). Fluorescence microscopy analysis at 72 h postinfection revealed infection efficiencies exceeding 76% in both groups (Figure 1A,B). The qRT‐PCR results showed that EBLN1 mRNA expression in the silenced group was reduced to 41% and 40% of the control and negative groups, respectively (*p* < 0.001; Figure 1C).

### 3.2 EBLN1 Silencing Suppresses the Proliferation of U‐87 MG Cells

**FIGURE 2:**
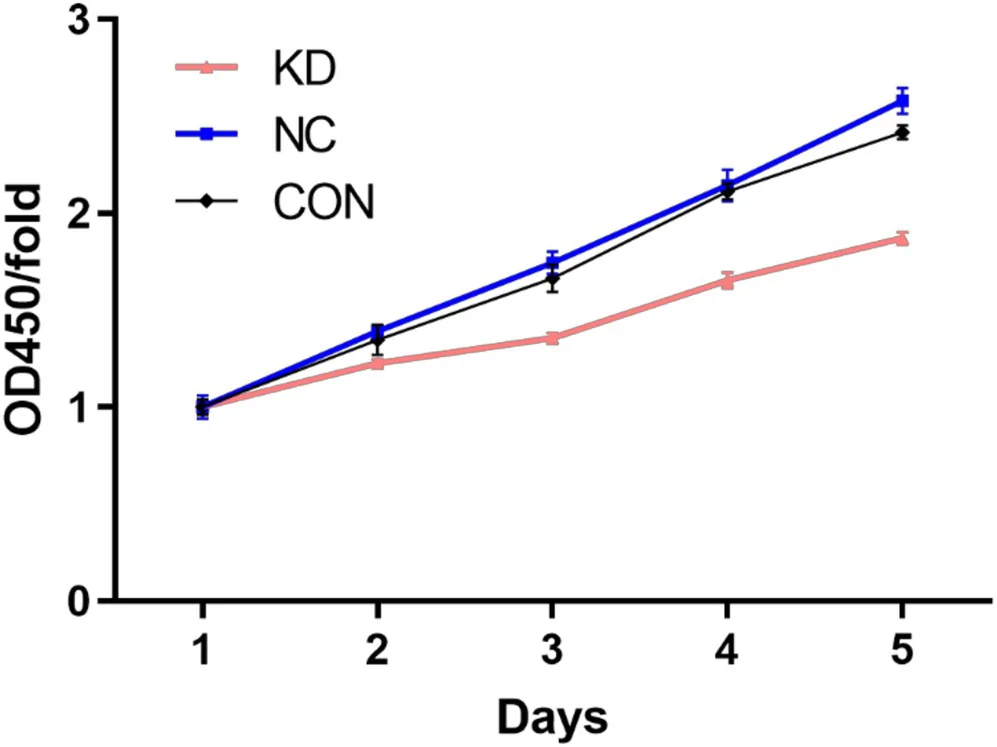
EBLN1 silencing suppresses the proliferation of U‐87 MG cells. CON, uninfected group; KD, EBLN1‐silenced group; NC, negative control group.

Based on previous findings that EBLN1 silencing suppresses oligodendrocyte progenitor (OL) cell proliferation, we investigated whether this effect is conserved in U‐87 MG cells. Using the CCK‐8 assay, we generated growth curves to assess cell proliferation dynamics. Our results revealed that EBLN1 silencing induced a significant reduction in U‐87 MG cell growth as early as the second day post‐infection (*p* < 0.05; Figure 2).

### 3.3 EBLN1 Silencing Induces the Apoptosis of U‐87 MG Cells

**FIGURE 3:**
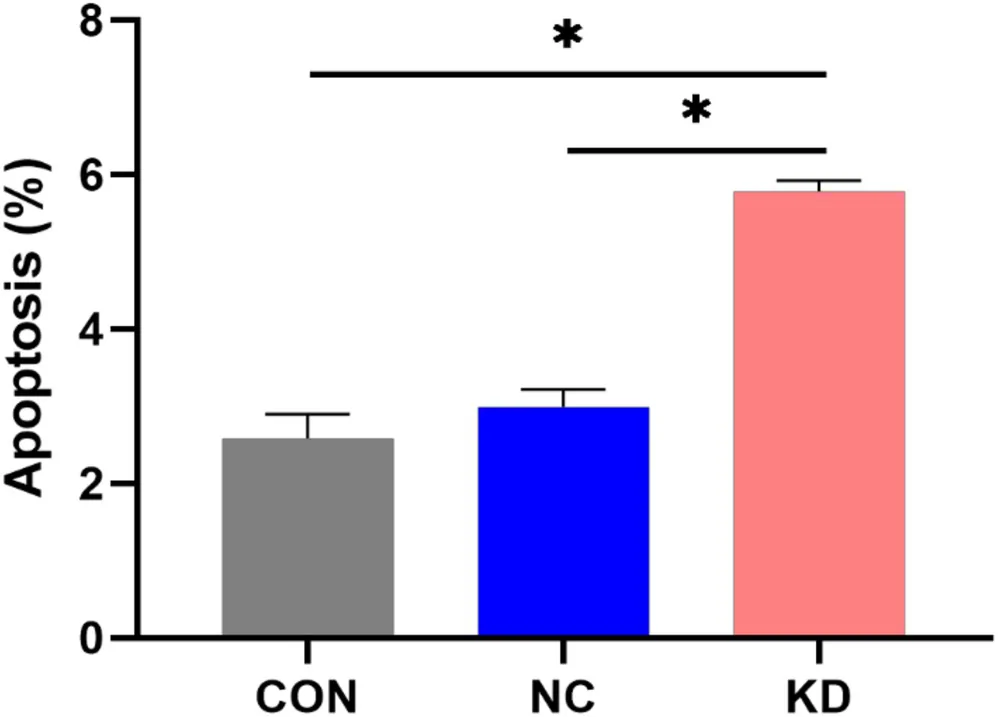
EBLN1 silencing increases the apoptosis of U‐87 MG cells. CON, uninfected group; KD, EBLN1‐silenced group; NC, negative control group. *p < 0.001.

To investigate the effects of EBLN1 silencing on apoptosis in U‐87 MG cells, we collected uninfected control and EBLN1‐silenced U‐87 MG cells, which were stained with annexin V‐APC. Apoptosis levels were subsequently quantified via flow cytometry. Results showed that at 72 h postinfection, the apoptosis rate of EBLN1‐silenced U‐87 MG cells reached 5.78% ± 0.14%, significantly higher than the uninfected group (2.58% ± 0.31%) and negative control group (2.99% ± 0.23%) (*p* < 0.001; Figure 3).

### 3.4 EBLN1 Silencing Increases DSBs in U‐87 MG Cells

**FIGURE 4:**
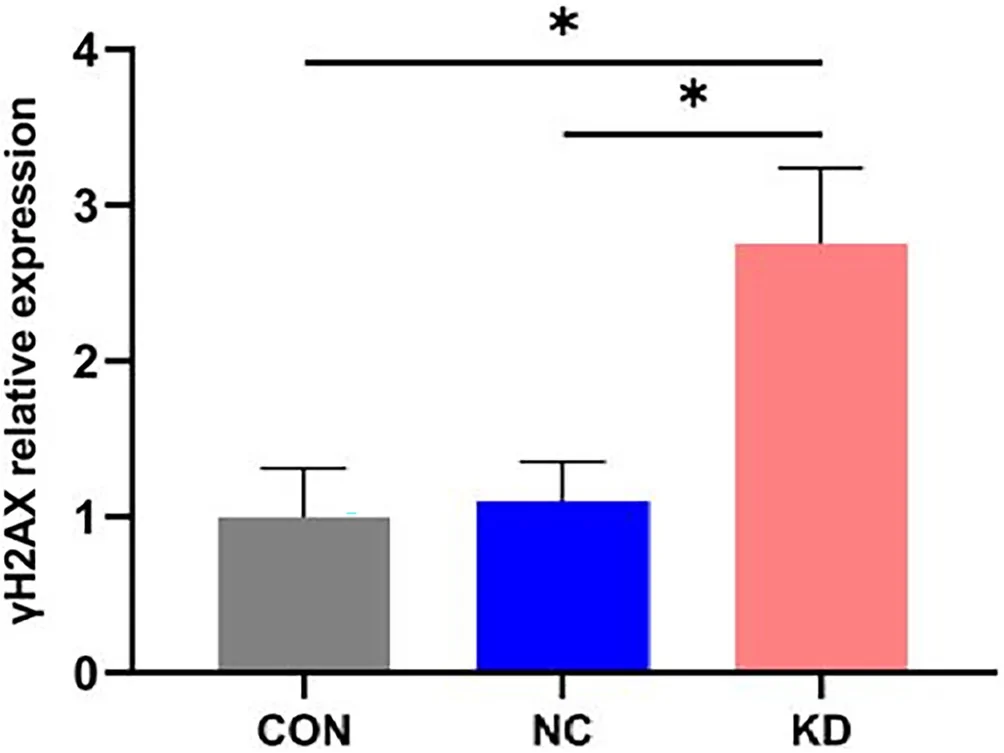
EBLN1 silencing increases DSBs in U‐87 MG cells. DSB levels were determined using ELISA for γH2AX. CON, uninfected group; KD, EBLN1‐silenced group; NC, negative control group. *p < 0.001.

Gamma H2AX (γH2AX) is the phosphorylated form of histone H2A and serves as the gold standard for estimating DSB levels [18]. To assess the effects of EBLN1 silencing on DSB levels in U‐87 MG cells, we measured γH2AX levels by ELISA. The results showed that the relative expressions of γH2AX in the uninfected group, the negative control group, and the EBLN1‐silenced group were 1.00 ± 0.32, 1.10 ± 0.25, and 2.75 ± 0.49, respectively. EBLN1‐silenced U‐87 MG cells exhibited a remarkable increase in the relative expression of γH2AX (*p* < 0.001; Figure 4).

### 3.5 EBLN1 Silencing Alleviates DSB Repair Response in U‐87 MG Cells

**FIGURE 5:**
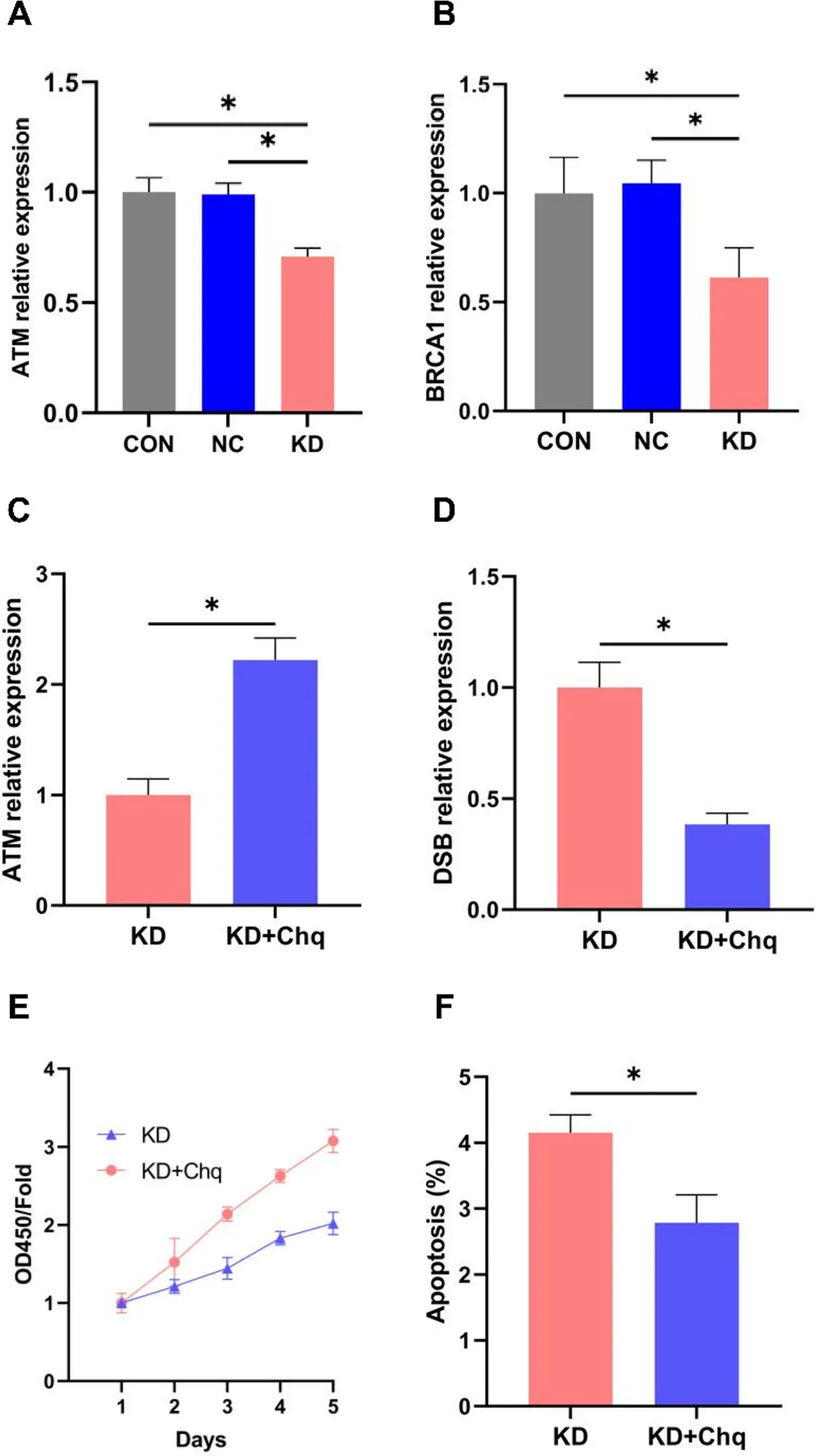
EBLN1 silencing inhibits the DSB repair factors in U‐87 MG cells. Reactivation of AMT reverses the malignant phenotype of EBLN1‐silenced U‐87 MG cells. (A) The levels of ATM were detected by ELISA; (B) the levels of BRCA1 were detected by ELISA; (C) the treatment with chloroquine (Chq) reactivated ATM; (D) the treatment with Chq decreased the DSB levels; (E) the treatment with Chq promoted cell proliferation; (F) the treatment with Chq suppressed apoptosis. CON, uninfected group; KD, EBLN1‐silenced group; KD + Chq, chloroquine‐treated EBLN1‐silenced group; NC, negative control group. *p < 0.001.

Based on our findings above, we hypothesized that the observed alterations in biological processes and elevated DSBs in EBLN1‐silenced U‐87 MG cells might arise from defective DDR. To investigate this mechanism, we quantified protein levels of ATM and BRCA1 via ELISA, both of which are critical for DDR.ATM is a member of the phosphatidylinositol 3‐kinase‐related kinase (PIKK) family, and phosphorylates H2AX and BRCA1 during DDR activation triggered by DSBs [19]. Compared to the uninfected group and the negative control group, the relative expression levels of ATM and BRCA1 in EBLN1‐silenced U‐87 MG cells were significantly reduced (ATM: 1.00 ± 0.07 and 1.00 ± 0.05 vs. 0.71 ± 0.04; BRCA1: 1.00 ± 0.17 and 1.04 ± 0.11 vs. 0.61 ± 0.14) (*p* < 0.001; Figure 5A,B).

### 3.6 Reactivation of AMT Reverses the Cellular Processes of EBLN1‐Silenced U‐87 MG Cells

To determine whether EBLN1 silencing alters cellular processes via the DDR pathway, we treated EBLN1‐silenced U‐87 MG cells with 25 μmol/L chloroquine (Chq). The results showed that after 24 h of Chq treatment, ATM was reactivated, which had been previously inhibited by EBLN1 silencing (*p* < 0.001 vs. control; Figure 5C). Meanwhile, the DSB levels in the treated cells were obviously decreased (*p* < 0.001 vs. control; Figure 5D). Furthermore, subsequent analyses found that compared to untreated cells, the treated cells' proliferation was significantly increased (*p* < 0.001; Figure 5E), while apoptosis was markedly suppressed (*p* < 0.001; Figure 5F). These findings indicated that ATM‐mediated DDR played a key role in cell proliferation and apoptosis regulated by EBLN1 in U‐87 MG cells.

## 4 Discussion

Tumorigenesis is the result of a series of complex cellular biochemical events, including DNA damage and repair. DNA damage frequently occurs due to endogenous and exogenous sources. Upon DNA damage, the DDR is immediately triggered to maintain cellular homeostasis and preserve genome integrity [20, 21]. The severity of DNA damage and the activity of DDR jointly determine the fate of DNA‐damaged cells, such as cell cycle arrest, apoptosis, or tumor cell formation [22]. DNA damage includes several types, with DSB representing the most severe DNA lesion. Previous studies have shown that DSB is closely related to the pathology of GBM and its resistance to chemotherapy treatment [6, 23, 24].

BoDV‐1 is a highly neurotropic RNA virus that causes severe encephalitis in human beings [25, 26, 27]. The BoDV‐1 genome encodes at least six viral proteins: nucleoprotein (N), phosphoprotein (P), X protein (X), matrix protein (M), glycoprotein (G), and polymerase (L) [11, 28, 29]. Human EBLN1 has a high homology to the BoDV‐1 N gene, and its functions are unclear. In this study, we found that silencing of EBLN1 increased DSB levels in U‐87 MG cells, which is consistent with the accumulation of DSBs in EBLN1‐depleted cells, such as HCT116 colorectal cancer cells and HeLa cervical cancer cells [15]. However, a recent study showed that expression of the BoDV‐1 N protein increased DSB levels in primary neurons [16]. These paradoxical results suggest that EBLN1 may function differently in normal and malignant cells. Moreover, despite having an intact open reading frame (ORF), EBLN1 was only verified at the transcript level. Thus, unlike the BoDV‐1 N protein, EBLN1 may function as an ncRNA in regulating DSB repair in U‐87 MG cells. In addition, whether EBLN1 has the same effects on DSB in normal and malignant cells needs further investigation.

Deficiency in EBLN1 expression could alter multiple cellular behaviors, including cell proliferation, cell cycle, and apoptosis. Previous studies have demonstrated that EBLN1‐depleted cell proliferation is suppressed and that the majority of cells reside in the G2/M phase of the cell cycle [11, 15]. In this study, we also observed that, compared to control cells, EBLN1‐silenced U‐87 MG cells showed remarkably lower proliferation from Day 3 to Day 5 after infection and a higher apoptosis ratio. These results confirm that EBLN1 plays a notable role in regulating the behaviors of U‐87 MG cells. In addition, EBLN1‐silenced U‐87 MG cells displayed more prominent antiproliferative effects than pro‐apoptotic effects, which may be due to increased DNA damage. On the one hand, DNA‐damaged cells should arrest cell cycle progression or exit the cell cycle to allow time for repair. On the other hand, DNA damage resulting from EBLN1 silencing may not be severe enough to trigger massive apoptosis in U‐87 MG cells.

DDR is a complex process that involves DNA repair and cell cycle checkpoint control, and it determines the fate of DNA‐damaged cells, including cell cycle arrest, apoptosis, or senescence [6]. DSBs are primarily repaired via the following two canonical pathways: homologous recombination (HR) and nonhomologous end joining (NHEJ). ATM and DNA‐PKcs are considered the central regulators in the HR and NHEJ pathways, respectively. ATM and DNA‐PKcs belong to the family of serine/threonine protein kinases. Upon DSBs, ATM is recruited to DSB sites and activated by the Mre11‐Rad5‐Nbs1 (MRN) complex. Activated ATM phosphorylates a series of substrates, including ATM itself, histone H2AX, CHK2, p53, and BRCA1 [30, 31, 32, 33]. Differently, DNA‐PKcs is activated and recruited by the Ku complex, and DNA‐PKcs phosphorylates downstream substrates, including DNA‐PKcs itself, histone H2AX, p53, XRCC4, XLF, and PAXX [34, 35, 36].

Accumulating evidence has demonstrated that inhibition of ATM increases the sensitivity of GBM cells to chemotherapy and radiation, leading to more DSBs and more apoptosis [32, 33]. These studies suggested that the HR pathway may be dominant in DSB repair in GBM. Thus, we sought to determine whether EBLN1 silencing increases DSBs in U‐87 MG cells by altering DSB repair protein kinases in the HR pathway. Our results showed that EBLN1‐silenced U‐87 MG cells had lower ATM and BRCA1 activity than control cells. Furthermore, our results showed that ATM reactivation by chloroquine could promote cell proliferation, inhibit apoptosis, and decrease DSB levels in EBLN1‐silenced U‐87 MG cells. These findings primarily confirmed that EBLN1 acts as a critical controller of DSB repair and cellular processes by regulating the activity of ATM. However, in addition to ATM and BRCA1, DSB repair involves other protein kinases, such as DNA‐PKcs and ATR, as well as their substrates and signaling pathways. Thus, further studies are required to elucidate the underlying molecular mechanism.

Several limitations of this study should be acknowledged. First, all experiments were conducted using a single GBM cell line, which may limit the generalizability of the results to other GBM cell lines or patient‐derived primary tumor cells. Second, the mechanistic evidence linking EBLN1 to the ATM‐mediated DNA damage response pathway remains indirect in this study. Future research should aim to provide direct evidence of molecular interactions between EBLN1 and key components of the ATM‐mediated DNA damage pathway. Despite these limitations, this study offers preliminary evidence supporting the functional role of EBLN1 in GBM progression and establishes an experimental foundation for further in‐depth mechanistic investigations.

## 5 Conclusion

Our work demonstrates that EBLN1 plays a critical role in maintaining the malignant phenotypes of U‐87 MG GBM cells, possibly by regulating the activities of protein kinases involved in DSB repair pathways. Our results reveal the biological functions of EBLN1 in GBM and identify a new molecular mechanism in GBM tumorigenesis and treatment resistance.

## Author Contributions

**Jing Liang:** conceptualization, methodology, validation, supervision, writing – original draft, writing – review and editing. **Dongdong Zeng:** investigation, formal analysis. **Yujie Guo:** methodology, formal analysis, writing – review and editing. **Cuiyuan Jing:** resources, investigation, data curation. **Lin Tang:** data curation, resources, investigation. **Zhanpeng Luo:** methodology, formal analysis. **Peng He:** conceptualization, methodology, validation, supervision, writing – original draft, writing – review and editing.

## Funding

This study was funded by the Science and Technology Department of Sichuan Province (2020JDRC0139).

## Ethics Statement

The authors have nothing to report.

## Consent

The authors have nothing to report.

## Conflicts of Interest

The authors declare no conflicts of interest.

## Data Availability

The data that support the findings of this study are available from the corresponding author upon reasonable request.
